# Supplementary material for: Facile Aqueous-Phase Synthesis of Bimetallic (AgPt, AgPd, and CuPt) and Trimetallic (AgCuPt) Nanoparticles
Source: Materials (Basel). 2020 Jan 7;13(2):254. doi: 10.3390/ma13020254 (PMC7013979; doi:10.3390/ma13020254)
Supplement: Supplementary file 1 [file materials-13-00254-s001.pdf]

materials

MDPI

Supporting Information

Facile Aqueous-Phase Synthesis of Bimetallic (AgPt, AgPd, and CuPt) and Trimetallic (AgCuPt) Nanoparticles

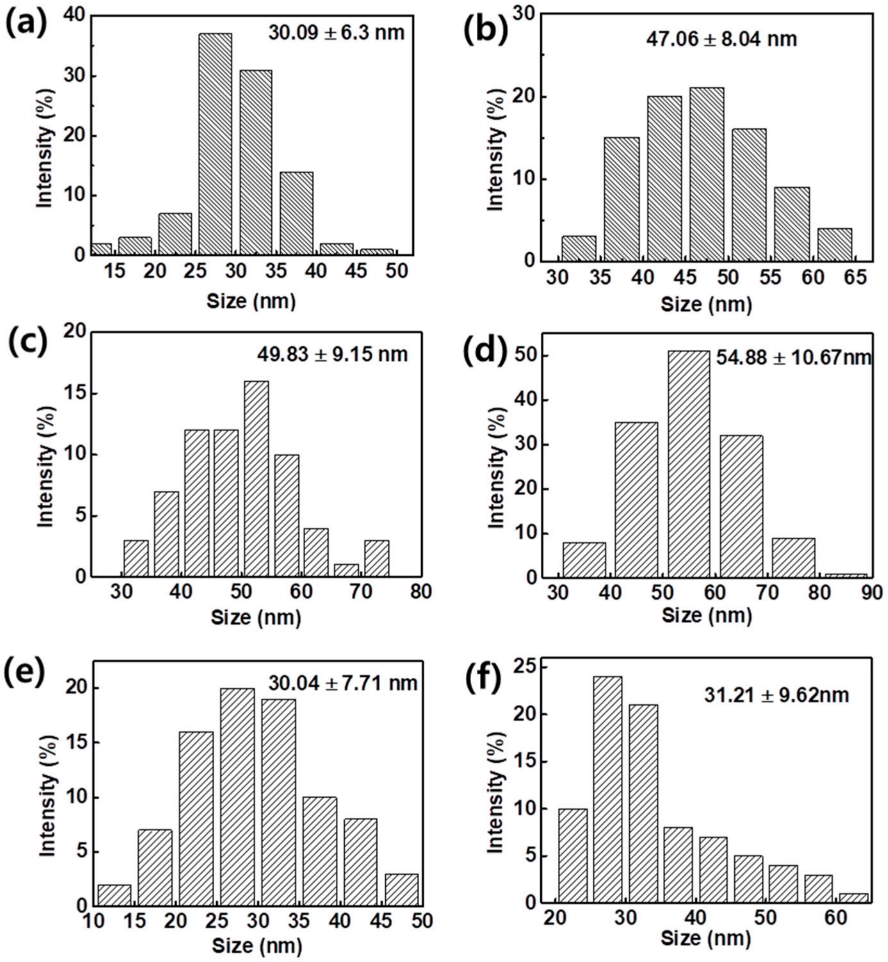

Figure S1. Size distributions of (**a**) AgPt-40, (**b**) AgPt-10, (**c**) AgPt-6.7, (**d**) AgPt-5, (**e**) AgPt-2, and (**f**) AgPt-1, respectively.

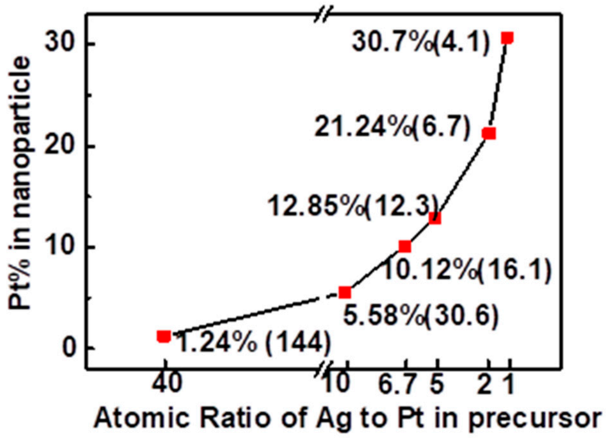

Figure S2. Pt percent in the AgPt nanoparticles synthesized at different atomic ratio of Ag to Pt in precursor. (The atomic ratio of Ag to Pt in nanoparticles is shown in the brackets).

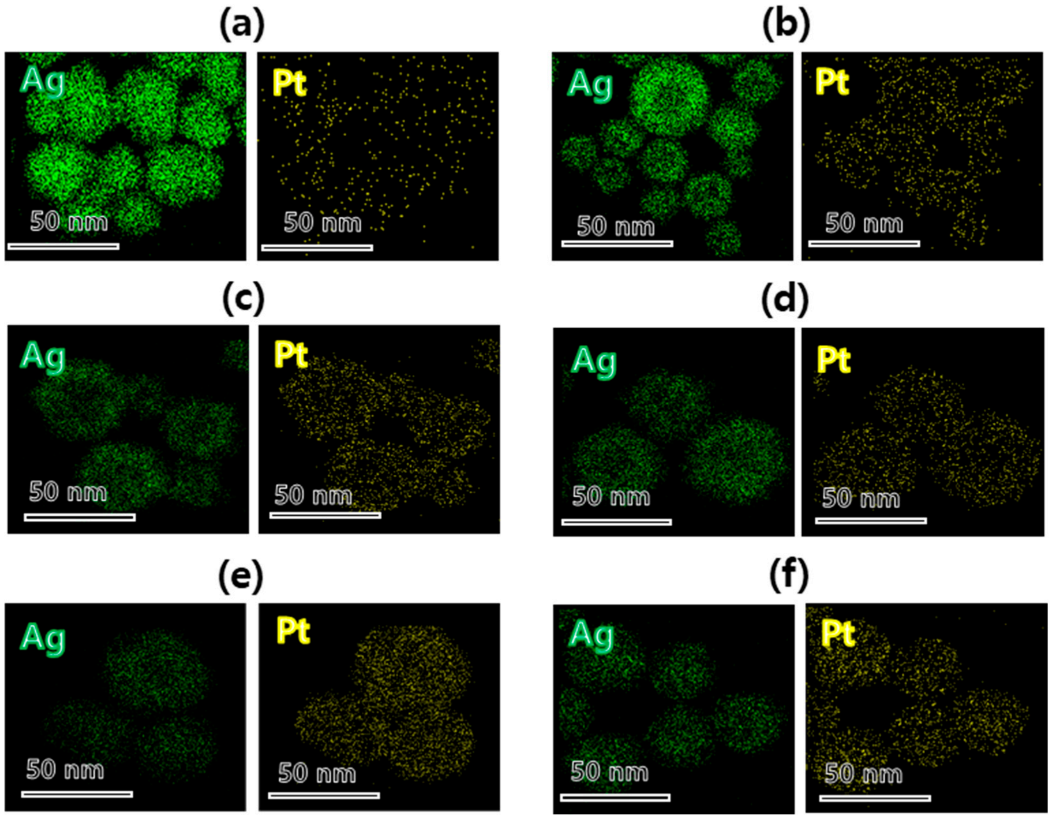

Figure S3. EDS mapping images of (**a**) AgPt-40, (**b**) AgPt-10, (**c**) AgPt-6.7, (**d**) AgPt-5, (**e**) AgPt-2, and (**f**) AgPt-1, respectively.

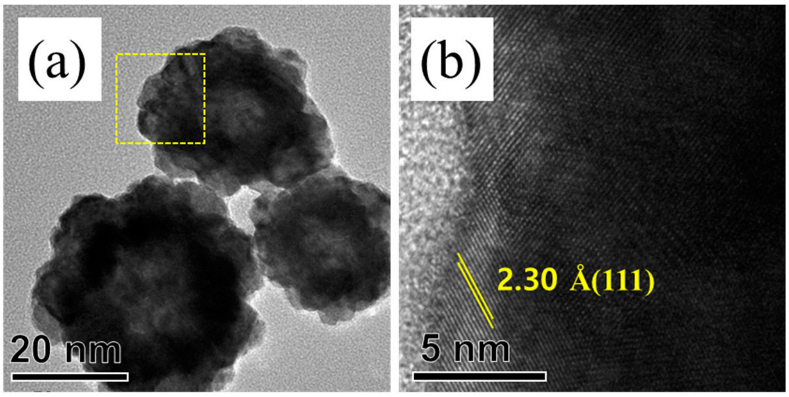

Figure S4. HRTEM imgaes of AgPt-6.7.

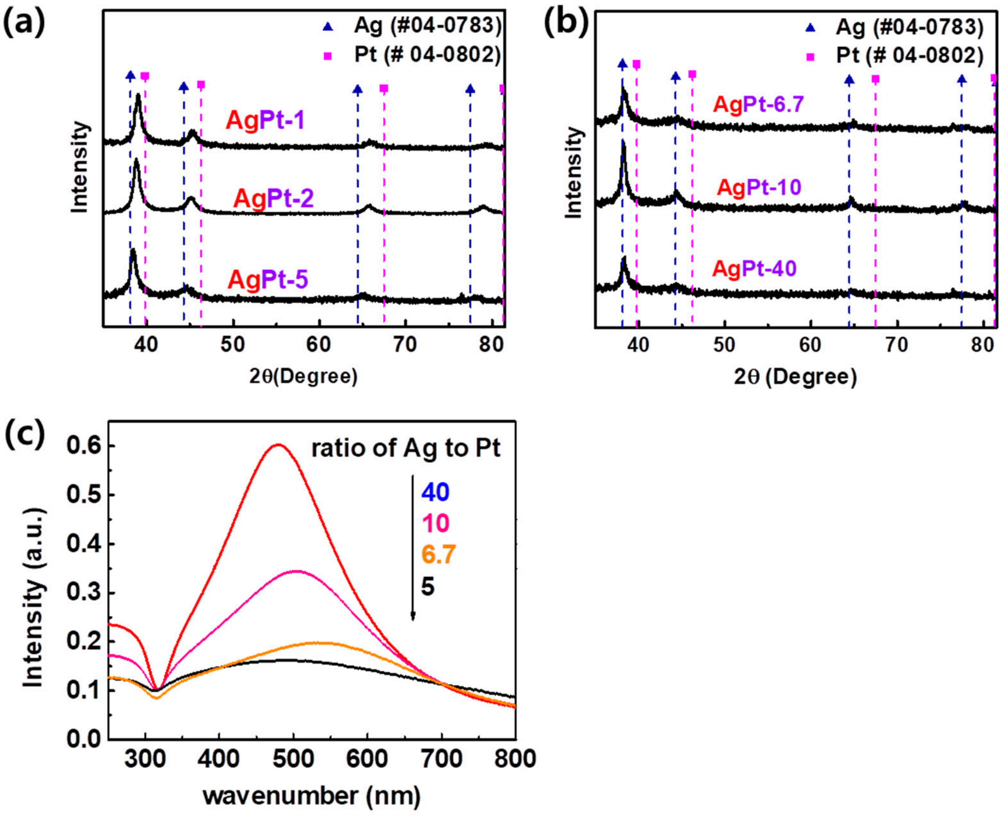

FigureS 5. (**a**) and (**b**) the XRD patterns, (**c**) UV-vis spectrum of as-prepared AgPt nanoparticles.

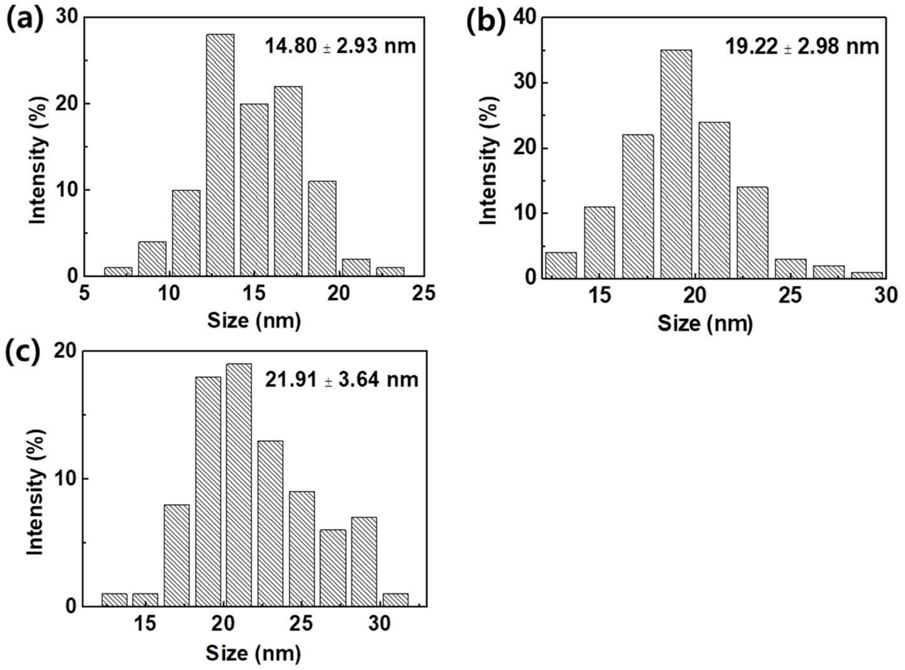

Figure S6. Size distributions of (a) AgPd-1, (b) AgPd-0.8, and (c) AgPd-0.66, respectively.

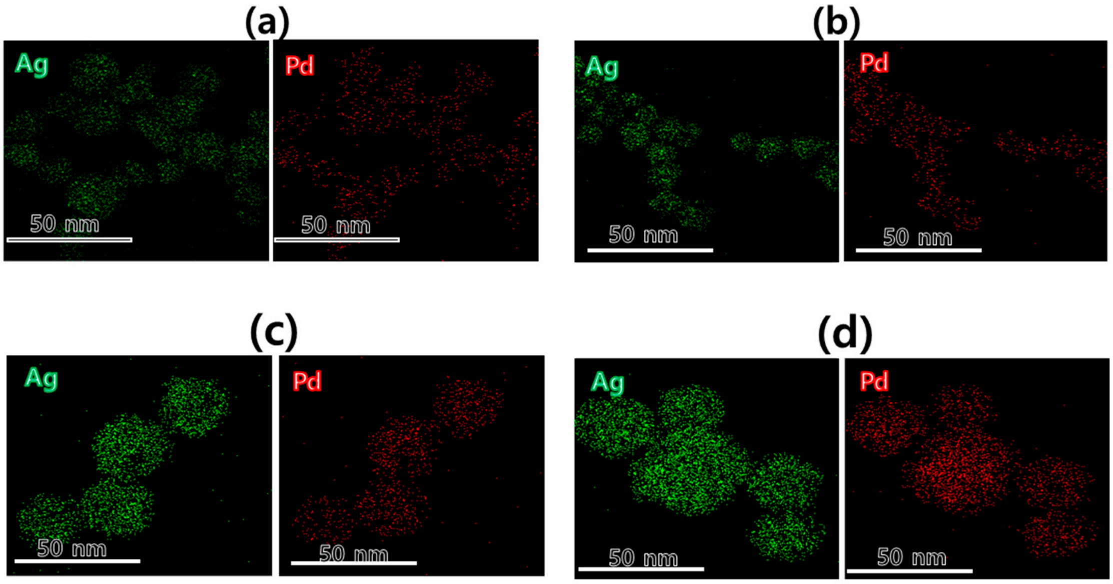

Figure S7. EDS mapping images of as-prepared AgPd nanoparticles with different Ag/Pd atomic ratio. The Ag/Pd atomic ratios were (a) 1.3, (b) 1, (c) 0.8, and (d) 0.66, respectively.

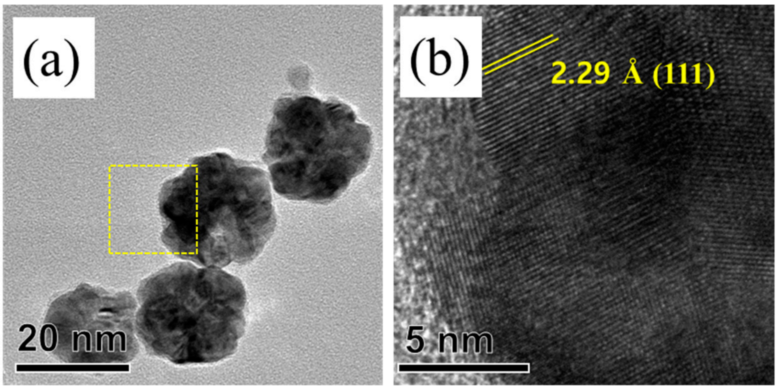

Figure S8. HRTEM imgaes of AgPd-1.

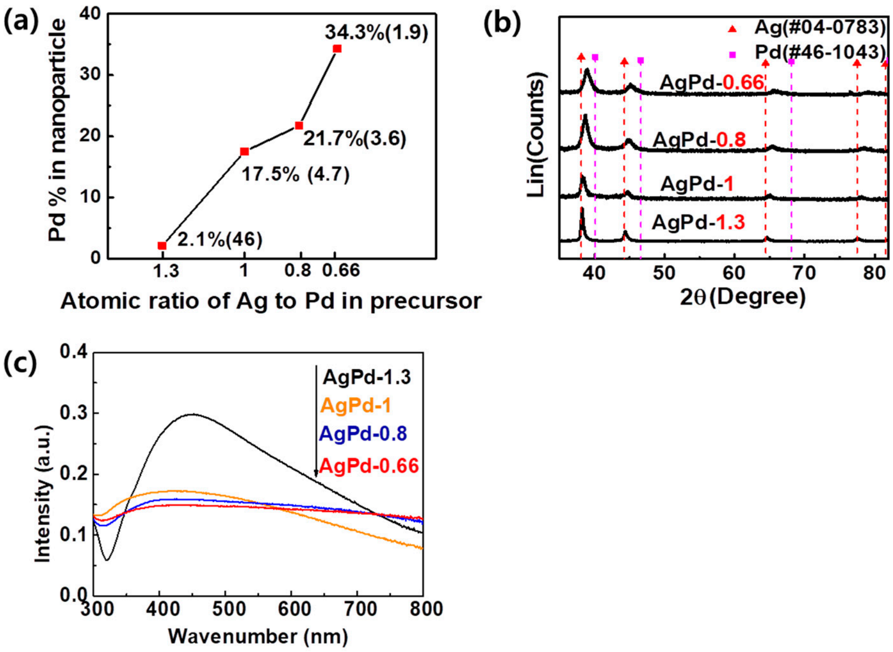

Figure S9. (a) Pd percent in the AgPd nanoparticle obtained at different atomic ratio of Ag to Pd in precursor. (The atomic ratio of Ag to Pd in the nanoparticles is shown in the brackets). (b) XRD patterns and (c) UV-vis spectrum of the AgPd nanoparticles.

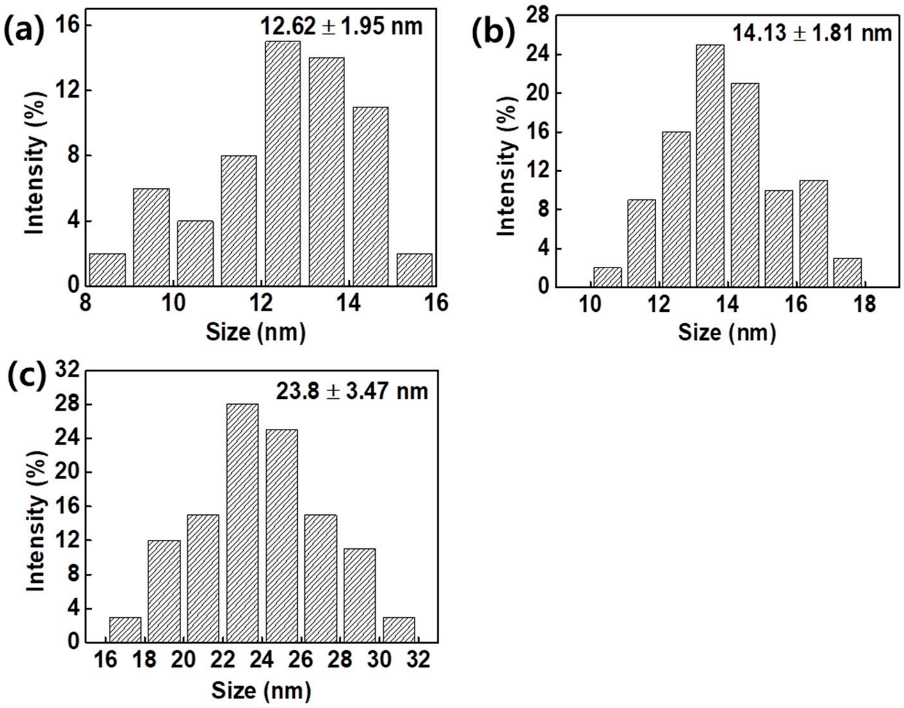

Figure S10. Size distributions of as-prepared CuPt nanoparticles (a) CuPt-20, (b) CuPt-10, (c) CuPt-4.

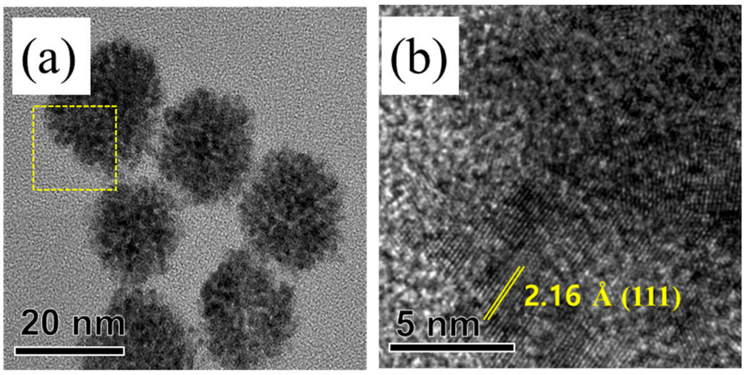

Figure S11. HRTEM images of CuPt-10.

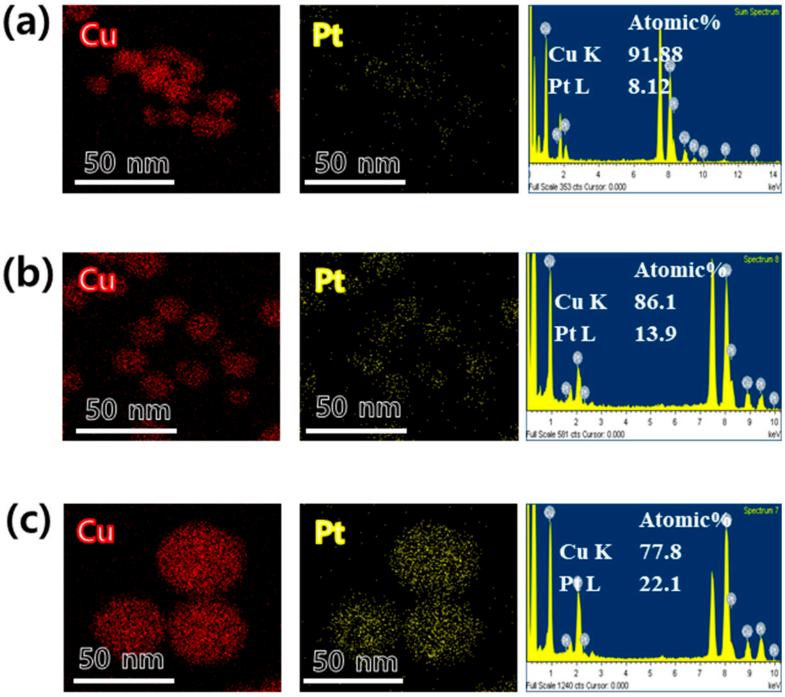

Figure S12. EDS mapping images of as-prepared CuPt nanoparticles (a) CuPt-20, (b) CuPt-10, and (c) CuPt-4.

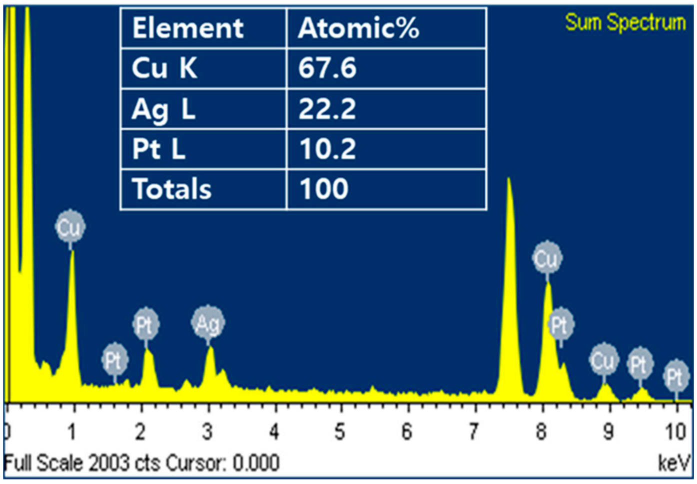

Figure S13. EDS data of atomic ratio Cu:Ag:Pt in AgCuPt nanoparticles.

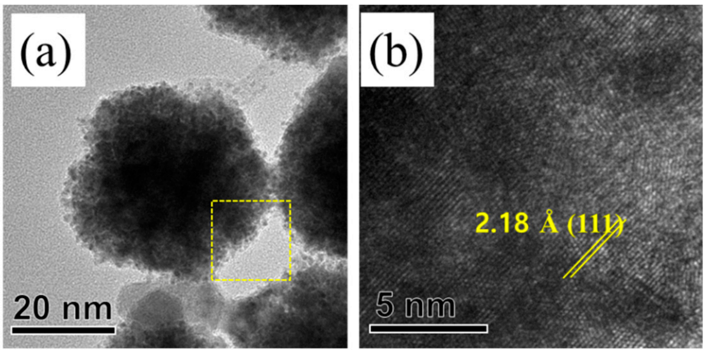

Figure S14. High resolution TEM (HRTEM) images of AgCuPt.

Table S1. Yield of each component in each multi-metallic nanoparticles.

Sample	Yield (%)		Pt% in NPS	Sample	Yield (%)		Pd% in NPS
AgPt-1	Pt	23.8%	30.7%	AgPd-0.66	Pd	31.8%	34.3%
	Ag	97.3%			Ag	97.8%	
AgPt-2	Pt	29.2%	21.24%	AgPd-0.8	Pd	20.1	21.7%
	Ag	96.8%			Ag	96.9%	
AgPt-5	Pt	38.4%	12.85%	AgPd-1	Pd	21.3%	17.5%
	Ag	96.9%			Ag	97.4%	
AgPt-6.7	Pt	41.0%	10.12%	AgPd-1.3	Pd	2.9%	2.1 %
	Ag	97.3%			Ag	95.9%	
AgPt-10	Pt	30.7	5.58%	CuPt-20	Pt	83.1%	-
	Ag	95.9%			Cu	34.0%	-
AgPt-40	Pt	26.7.7%	1.24%	CuPt-10	Pt	81.5%	-
	Ag	96.89%			Cu	52.1%	-
AgCuPt	Pt	88.7%	-	CuPt-4	Pt	82.1%	-
	Ag	96.4%			Cu	77.5%	-
	Cu	58.7%					-

Table S2. Zeta potential values of each nanoparticles.

Sample	Z-potential (mV)	Sample	Z-potential (mV)
AgPt-1	23.7	AgPd-0.66	25.9
AgPt-2	21.8	AgPd-0.8	28.3
AgPt-5	24.5	AgPd-1	27.2
AgPt-6.7	22.9	AgPd-1.3	26.7
AgPt-10	20.5	CuPt-4	28.1
AgPt-40	21.3	CuPt-10	27.4
AgCuPt	20.6	CuPt-20	29.6

© 2020 by the authors. Submitted for possible open access publication under the terms and conditions of the Creative Commons Attribution (CC BY) license (http://creativecommons.org/licenses/by/4.0/).
